# Analysing All-Optical Random Bit Sequences Using Gap-Based Approaches †

**DOI:** 10.3390/s24144474

**Published:** 2024-07-11

**Authors:** Christoph Lange, Andreas Ahrens, Jasmeet Singh, Olaf Grote

**Affiliations:** 1 Faculty 1: School of Engineering—Energy and Information, Hochschule für Technik und Wirtschaft Berlin (University of Applied Sciences), 10313 Berlin, Germany; 2 Faculty of Engineering, Hochschule Wismar (University of Applied Sciences: Technology, Business and Design), 23966 Wismar, Germany; andreas.ahrens@hs-wismar.de (A.A.); jasmeet.singh@hs-wismar.de (J.S.); 3 Escuela Técnica Superior de Ingeniería y Sistemas de Telecomunicación (ETSIST), Universidad Politécnica de Madrid, Campus Sur, Calle Nikola Tesla s/n, 28031 Madrid, Spain; olaf.grote@alumnos.upm.es

**Keywords:** pseudo-random binary sequences, gold sequences, *m*-sequences, all-optically generated bit sequences, gap density function, gap distribution function, burstiness, independently and identically distributed random variables

## Abstract

Quantum mechanical phenomena are revolutionizing classical engineering fields such as signal processing or cryptography. When randomness plays an important role, like in cryptography where random bit sequences guarantee certain levels of security, quantum mechanical phenomena allow new ways of generating random bit sequences. Such sequences have a lot of applications in the communication sector, e.g., regarding data transmission, simulation, sensors or radars, and beyond. They can be generated deterministically (e.g., by using polynomials, resulting in pseudo-random sequences) or in a non-deterministic way (e.g., by using physical noise sources like external devices or sensors, resulting in random sequences). Important characteristics of such binary sequences can be modelled by gap processes in conjunction with the probability theory. Recently, all-optical approaches have attracted a lot of research interest. In this work, an adaptation of the quantum key distribution setup is utilized for generating randomised bit sequences. The simulation results show that all-optically generated sequences very well resemble the theoretically ideal probability density characteristic. Additionally, an experimental optical setup is developed that confirms the simulation results. Furthermore, *m*-sequences show very promising results as well as Gold sequences. Additionally, the level of burstiness, i.e., the distribution of ones and zeros throughout the sequence, is studied for the different sequences. The results enable the finding that generator polynomials with concentrated non-zero coefficients lead to more bursty bit sequences.

## 1. Introduction

Since the pioneering work of Price and Green [1], random bit sequences have attracted a lot of attention for a broad range of applications, such as code division multiplexing-assisted data transmission schemes (e.g., [2]), radar communication [3,4] and sensor applications using fibre Bragg gratings [5]. Randomized bit sequences are of immense relevance in the context of information security and cryptography, where bit sequences form the basis of cryptographic keys [6]. Such random bit sequences are crucial elements to ensure the confidentiality, integrity, and authenticity of digital data and also play a role in generating ephemeral keys for digital signatures. Strong cryptographic keys are required to encrypt and decrypt data securely, and they need to be unpredictable and resistant to brute-force and replay attacks [7]. This requires a certain degree of randomness and independence for the random variables [8]. Effectively generated and utilized bit sequences are crucial in mitigating various security threats and vulnerabilities and in preventing deterministic behaviour. Criteria for measuring randomness of bit sequences are published, e.g., by the National Institute of Standards and Technology (NIST) [9].

In the proposal [10], Peter and Schindler divided the generation of random sequences into deterministic, non-deterministic, and non-physical classes. Deterministic algorithms take a short random sequence as an input value, called *seed*, and use it to generate an output bit sequence that appears random but is pseudo-random, i.e., algorithmically generated. The unpredictability of this class depends on the entropy and safety of the *seed*. Non-deterministic or physical true entropy sources generate randomness based on physical phenomena such as noise or quantum noise. Non-physical methods use the device environment, such as the file system, to generate random sequences with arbitrary data as an entropy source. For many applications, pseudo-random binary sequences, such as *m*-sequences [6,11] or Gold sequences [12] (also known as Gold codes), generated with the help of deterministic algorithms—like manipulating binary elements by using shift registers—show a sufficient level of randomness. Furthermore, they show the advantage that they can be generated by a computer software. Here, the unpredictability is based on the correct seed implementation or by using chaotic systems based on artificial neural networks (ANN) and ring oscillators, as described in [13].

In the field of sensor technologies, fibre optical sensors have attracted a lot of attention based on their inherent advantages such as small size, low weight, as well as robustness and resilience against electro-magnetic interference. These benefits make sensor systems a suitable and advantageous choice for many applications [14]. Based on the capability of multiplexing, fibre optical sensors are predestined for applications in the fields of structural health monitoring [15] and smart structures [16]. The most popular multiplexing schemes applied in sensor networks are wavelength-division multiplexing, time-division multiplexing, and optical frequency domain refractometry. Next to them a code-division multiplexing scheme was introduced in [5], whose performance strongly depends on the selected (pseudo-random) spreading sequence.

With the development of quantum computing, the potential of transmitting secure data based on quantum mechanics is widely being researched recently. A possible solution is represented by the quantum key distribution (QKD) as an innovative key exchange protocol, exemplified by the popular protocols BB84 (developed by Bennett and Brassard in 1984) [17] and E91 (developed by Ekert in 1991) [18], which focus on the security perspective.

Furthermore, all-optical software can be used to simulate QKD protocols as described in [19,20], or derivatives such as [21]. In this work, an adaptation of the QKD setup is utilized in order to simulate randomised bit sequences as QKD, which is based on microphysical phenomena that photons can attain either polarities or spins. Each polarised photon represents a single qubit [22]. The property of each photon is generated in this work via a pseudo-random number generator using OptiSystem [23]. The proposed all-optical setup works upon the laws of quantum mechanics [24]. Moreover, the generated bit sequence is based on QKD principles, which will produce high-quality random numbers under specific conditions [25,26]. In addition, the related simulation results are confirmed by means of an experimental setup.

For the application of the generated random binary sequences it is important that their elements are independently and identically distributed (IID) [27]. This means that all random variables, i.e., elements, are produced independently (the individual bits do not affect each other) and that they obey an identical probability distribution each (here, a discrete uniform distribution of non-zero and zero elements within a binary sequence).

The novelty of this paper is based on amalgamating an all-optical approach for generating random bit sequences with a newly derived and adapted gap-based method for describing binary sequences. The derived gap-based probability density functions serve as a foundation for analysing the obtained sequences. As an additional parameter the concentration of the non-zero elements is studied, leading to the burstiness definition. The gap-based approach provides a detailed analysis with regards to the IID characteristics on the binary sequence, where the commonly used NIST criteria only show one of the two outcomes, either pass or fail. An experimental setup has been constructed to verify the effectiveness of the all-optical setup approach. The experimental results confirm the IID hypothesis that has been formulated for the all-optical approach using the simulation results. The focus of this work, firstly reported in [28], is on persistently characterizing the randomness of any given binary sequence by applying the derived gap-based distribution and gap-based distribution function. Therefore, the gap-based approach allows a detailed insight into the inner structure of any given random binary sequence. The effectiveness of the proposed gap-based approach is analysed on different deterministic algorithms and cryptographic primitives. An optical system is designed and simulated based on the applications of quantum communication to evaluate the generated binary sequences with the provided gap-based approach. Furthermore, an experimental setup is also constructed based on the simulation model. Finally, the result from the simulation and experimental setups also verify the robustness and effectiveness of the proposed gap-based approach. The random bit generation in a broadband random optoelectronic oscillator is exemplarily demonstrated in [29], where the randomness of these sequences is verified by using NIST (National Institute of Standards and Technology) statistical tests. In [30] the NIST test suite is used to evaluate the quality of the investigated bit sequences.

The remaining parts of this paper are organized as follows: Section 2 introduces the proposed concept of gap-based modelling of binary sequences. The generation of binary sequences using an optical approach as well as other options are discussed in Section 3. The simulation results are analysed in Section 5 based on evaluation criteria introduced in Section 4. In Section 6, important findings are discussed. Finally, concluding remarks are provided in Section 7.

## 2. Gap-Based Modelling of Binary Sequences

### 2.1. Introduction and Principles

**Figure 1:**
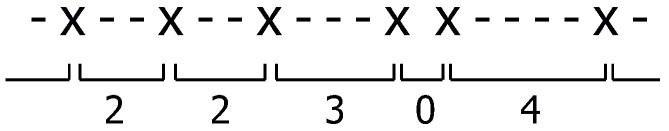
Modelling the intervals between non-zero elements by gaps (a non-zero element, represented by “x”, is separated by gaps of different lengths, represented by “-” and describing zero elements within a given binary sequence).

An approach for the investigation of the randomness of given binary sequences can be obtained by analysing the distribution of the gaps between neighbouring non-zero elements within a given binary sequence. Figure 1 shows how the gap structure of a given binary sequence can be obtained: the numbers below the structure consisting of the elements "x" and "-" represent the lengths of the gaps.

**Figure 2:**
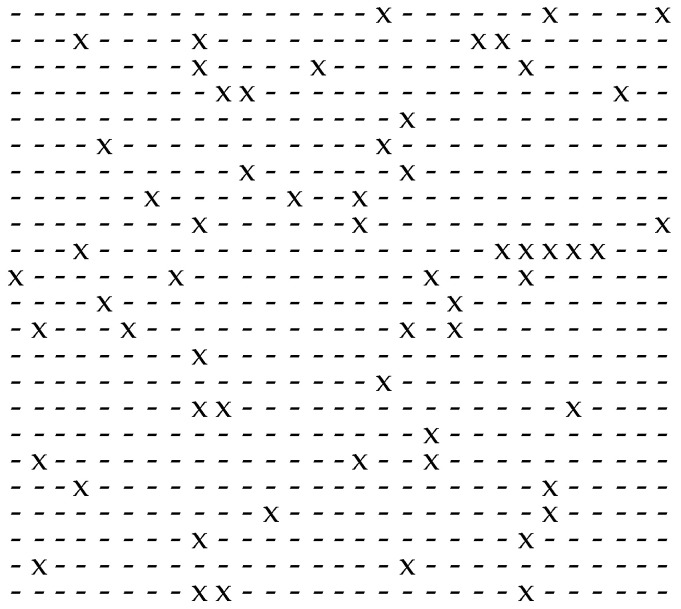
Separation of the non-zero elements, represented by “x”, surrounded by zero element, represented by “-”, within a given binary sequence in a non-bursty scenario (i.e., independently distributed non-zero elements).

**Figure 3:**
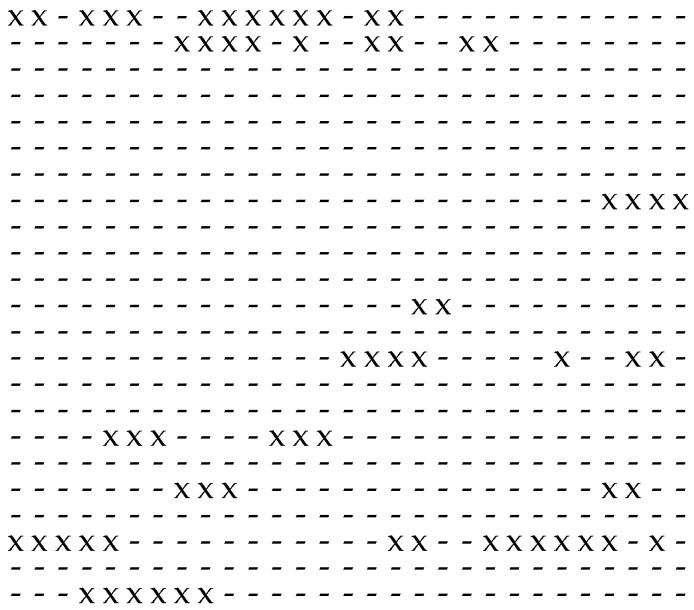
Separation of the non-zero elements, represented by “x”, surrounded by zero element, represented by “-”, within a given frame with bursty appearance of the non-zero element.

The distribution of the non-zero elements in a given binary sequence is illustrated in Figure 2 and Figure 3. Whereas in Figure 2 the non-zero elements are more independently distributed, in Figure 3 they appear burstily distributed. A preferable characteristic of random binary sequences is the IID property: transferring this IID characteristic to random bit sequences requires that the non-zero elements should appear more independently distributed, where long sequences of non-zero elements (i.e., bursts) should be avoided.

### 2.2. Mathematical Description

From this, the gap distribution function u(k), defined as the probability that a gap *Y* between two non-zero elements is greater than or at least equal to a given number of *k* (with k∈Z), i.e.,

(1) u(k)=P(Y≥k),

can be derived.

It is worth noting that in probability theory the distribution function is defined as the probability that a trial (random variable) *Y* takes on a value less than or equal to a number *k*, i.e., P(Y≤k). Next to the distribution function (often also referred to as cumulative distribution function) it is prevalent to use the complementary cumulative distribution function when the question appears in terms of how often a random variable *Y* is above a particular level *k*, i.e., P(Y>k). In contrast to these definitions, in this work a gap distribution function u(k) is used as defined in (1)—since this definition is usually applied in the context of gap-based analyses, e.g., in [31,32] and in the form of a gap distance distribution in [33]. Furthermore, in wireless communications error gap distribution functions (sometimes also referred to as error gap distance distributions) have been successfully applied to the optimization of communication systems as early as in the 1960s and 1970s [34].

Re-writing of u(k) leads to the gap density function v(k)=P(Y=k), which describes the probability of a gap *Y* equal to *k*:

$$\begin{array}{cc} u(k)= & \;\;v(k)+v(k+1)+v(k+2)+\cdots \\ u(k+1)= & \;\;v(k+1)+v(k+2)+\cdots \;. \end{array}$$

By calculating the difference between u(k) and u(k+1), the gap density function v(k)=P(Y=k) can be obtained as

(2) v(k)=u(k)−u(k+1).

For many applications, a uniform distribution of the non-zero elements in a sequence, for example in a bit stream, is a preferred characteristic, e.g., [11]. Such scenarios can be described solely by the element occurrence probability $p_{e}$, defining the probability that a given element in a sequence is non-zero—which equals the probability that a zero element occurs. When analysing a given bitstream $s=(s_{0},s_{1},\ldots ,s_{n-1})$ of finite length *n*, the element occurrence probability $p_{e}$ is defined as

(3) $$p_{e}=P\left(s_{\ell }=1\right)\;\mathrm{for}\;0\leq \;\ell \leq n-1\;.$$

Since binary sequences are considered in this work, the elements of the bitstream s can be interpreted as bits and the element occurrence probability merges into the bit occurrence probability (BOP).

With (1), the inner structure of such a bitstream s can be described by a sequence of gaps (as shown in Figure 1). Assuming that the bits of the sequence s are independently distributed (also referred to as non-bursty non-zero elements) the gap distribution function u(k) can be derived as a function of the BOP $p_{e}$ [35]. With the BOP $p_{e}$, the probability that a single element in the sequence is zero, is given by $\left(1-p_{e}\right)$. Therefore, the probability u(k)=P(Y≥k) that ≥k neighbouring elements in the bitstream are zero results in

(4) $$u\left(k\right)={\left(1-p_{e}\right)}^{k}\;0\leq k<\infty \;,$$

and can be approximated for small $p_{e}$ as

(5) $$u\left(k\right)\approx e^{-p_{e}\cdot k}\;.$$

Equation (4) is well-known in probability theory for non-bursty, i.e., independent, events [35] and is valid for any probability $p_{e}$. In an ideal scenario, a given bit sequence should follow either (4) or (5). The approximation of (5) is based on the Taylor series of the exponential function $e^{-\;x}$ for small *x*. The function $e^{-\;x}$ can be re-written with [36] as

(6) $$e^{-\;x}=1-x+\frac{x^{2}}{2}-\frac{x^{3}}{6}+\frac{x^{4}}{24}+\cdots \;,$$

and approximated by

(7) $$e^{-\;x}\approx 1-x\;$$

for small *x*.

Furthermore, using (2) with (4), the gap density function v(k) is obtained as

(8) $$v\left(k\right)={\left(1-p_{e}\right)}^{k}\cdot p_{e}\;.$$

From (8) follows with $v\left(0\right)=p_{e}$ and with $p_{e}=0.5$ that the probability that a given element of the sequence is non-zero equals the probability that a zero element is generated. This implies a probability of 0.5 that after a non-zero element the following element is non-zero as well. Furthermore, as no memory between gaps exists, the gap lengths within a given binary sequence are considered as independent from each other.

**Figure 4:**
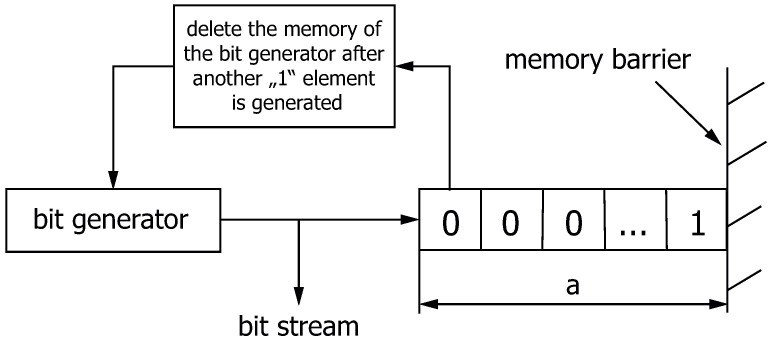
Generating gap structures with independent gaps.

Figure 4 illustrates the theoretical model of the generation of gap processes with independent gaps. There is only one “1” element in the shift register at the earliest position. The length of the shift register is extended step by step with each cycle until the next “1” element is generated. In the next step, the shift register has the length a=1 and is filled with the “1” element that has just been generated. The memory only goes as far as the last “1” element and the gap distribution function, defined in (1), can be used to describe the distribution of the gaps.

**Figure 5:**
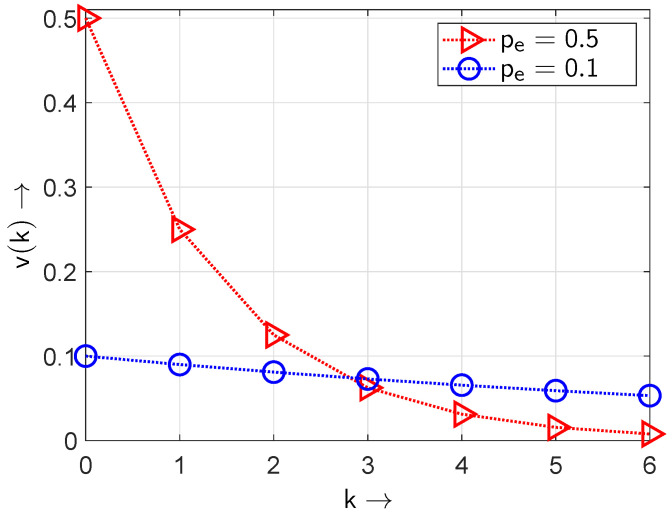
Gap density function v(k) for different bit occurrence probabilities $p_{e}$.

Figure 5 shows the obtained gap density functions v(k) for different values of $p_{e}$ taking (8) into account.

**Figure 6:**
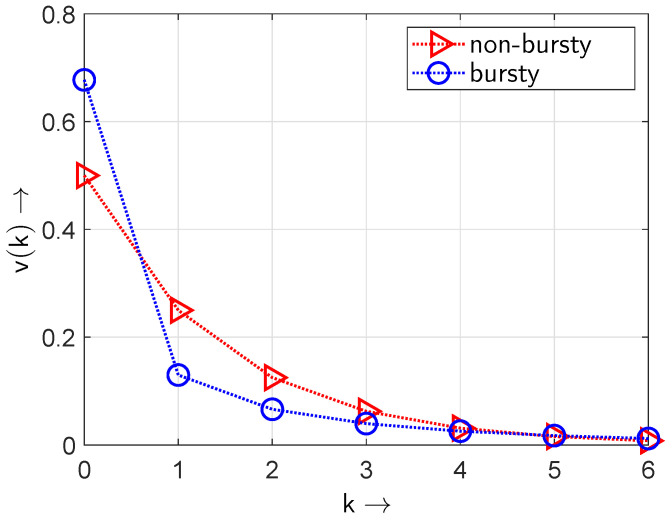
Gap density function v(k) for bursty and non-bursty sequences at a BOP of $p_{e}=0.5$.

Whereas the gap density function v(k) defined in (8) is solely applicable for independent (non-bursty) events, the bursty appearance of the non-zero element in the sequence requires gap distribution functions with a least two parameters introduced by Weibull [37] or Wilhelm [33]. The changes in the gap density function v(k) assuming an element occurrence probability $p_{e}=0.5$ are illustrated in Figure 6. Under bursty conditions shorter gaps appear with a higher probability at the cost that longer gaps now appear with a lower probability—which will destroy the IID property with v(0)=0.5.

## 3. Generation of Binary Sequences

### 3.1. Introduction

As discussed in the previous section, the ideal binary sequence should follow u(k) as derived in (4) or (5) or v(k) according to (8), respectively, in terms of probability distribution. Now, in this section some practical realizations are studied. This section at first introduces the generation of all-optical bit sequences. After that, other options are shortly reviewed, e.g., *m*-sequences and Gold sequences. Finally, some cryptographic primitives are analysed.

### 3.2. All-Optically Generated Bit Sequences

In this subsection the simulation concept for all-optically generated random bit sequences is introduced and the experimental setup for their practical verification is described.

#### 3.2.1. Simulation Setup

The proposed all-optical setup works upon the laws of quantum mechanics, where each bit represents a single photon. The stream of polarised photons is propagated across an optical fibre within the simulation. At the receiver, the physical orientation of each photon is determined using a polarised filter. The polarisation state of each photon is modified by using an optical switch, which is triggered with a pseudo-random number generator. Since other optical components are also deterministically mapped in the optical simulation, the purely optical simulation is mapped to a mathematical simulation environment such as Matlab R2022a [38].

**Figure 7:**
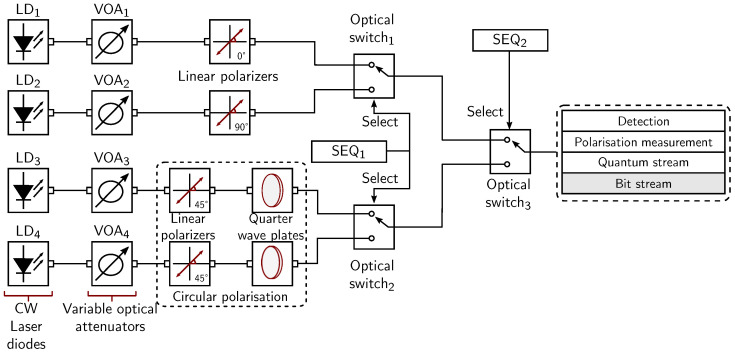
Setup of quantum-based binary sequence generator in OptiSystem software.

An optical simulation setup is constructed for generating a bit stream by using OptiSystem software [23], which is illustrated in Figure 7. This bit stream generation process starts by utilizing four laser diodes (LD) with an operating wavelength of 1552 nm. A single photon is excited on each laser diode due to the quantum nature of the light. Subsequently, variable optical attenuators (VOAs) are incorporated to control the transmission power throughout this setup. Afterwards, the polarisation state of each photon is modified using circular and linear polarizers. The first two branches with laser diodes, i.e., LD_1_ and LD_2_, provide horizontally and vertically linearly polarised states to the photons. These two polarisation states from the linear polarizers are supplied to the first optical switch, which selects one of the two linear polarisation states. The selection criteria of the polarisation state depend upon the provided sequence (SEQ_1_), which should ideally be IID. The rule-based selection process chooses a linear 0° state when a binary 0 is encountered from the SEQ_1_. Similarly, a linear 90° state is selected when a binary 1 arrives at the selected input of the first optical switch. The circular polarisation states are generated using linear polarizers and quarter-wave plates. The photons arriving from LD_3_ and LD_4_ are firstly linearly polarised at 45°. Thus, the electric field distribution of the linearly polarised photons consists of components along the *x*-axis and *y*-axis, which propagates through the quarter-wave plate. Since the refractive indexes of the quarter-wave plate are distinct along the *x*-axis and *y*-axis, the photon undergoes left or right circular polarisation. The two different circular polarisation states are created by using an opposite axis of the quarter-wave plate. Similarly, circular left and right polarisation states are chosen with a binary 0 and a binary 1 at the select input of the second optical switch, respectively. In the following step, another selection process is conducted using the third optical switch, which uses a second sequence SEQ_2_. The implemented rule for the selection states that a linear polarisation state is selected with a binary 0 and a circular polarisation state will be selected with a binary 1. In the last step, the received polarisation state undergoes detection and polarisation measurement mechanisms. Consequently, a quantum stream is obtained, which contains the received photons with different polarisation states. Afterwards, the quantum stream is converted into a bit stream to form the desired random binary sequence.

#### 3.2.2. Experimental Setup

**Figure 8:**
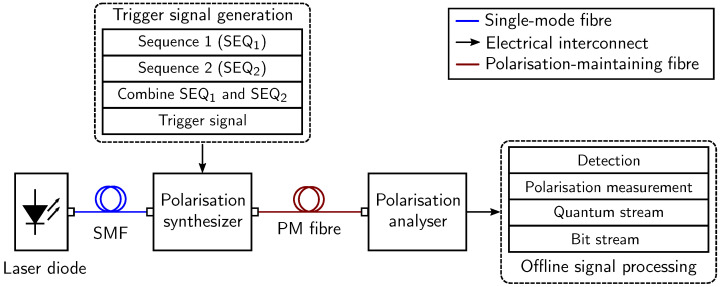
An equivalent experimental block diagram of the all-optical setup.

The concept of all-optical setup is translated into a polarisation-based random number generator, which is dependent upon generating and analyzing the polarisation. Thus, an equivalent experimental setup of the all-optical approach is developed in order to confirm the concept of Figure 7, which is illustrated in Figure 8. Firstly, a laser diode is utilized to produce continuous and non-polarised light with a maximum transmit power of −3.68 dBm operating at a wavelength of 1550 nm. The laser diode is connected with the polarisation synthesizer using a single-mode fibre (SMF) of 1m length. A specific state of polarisation (SOP) is created using the polarisation synthesizer, which is controlled by the trigger signal. The construction of the trigger signal includes a combination of two randomly generated binary sequences namely SEQ_1_ and SEQ_2_. For instance, the binary output of the first sequence is used to determine two out of four SOPs. A detailed description of the selected SOP using SEQ_1_ and SEQ_2_ is provided in Table 1, where linear SOP, i.e., horizontal and vertical, and circular SOP, i.e., right circular and left circular, are shown. The vertical polarisation and the right circular polarisation are chosen with bit ’1’ encountered on SEQ_1_. Consequently, only one polarisation state is chosen with SEQ_2_ in the next step. For instance, either linear or circular polarisation are selected with bit ’0’ or bit ’1’ of SEQ_2_, respectively. While creating a trigger signal, the generation of SEQ_1_ and SEQ_2_ are considered to be initial steps. The characteristics of *m*-sequences are incorporated for SEQ_1_.

**Figure 9:**
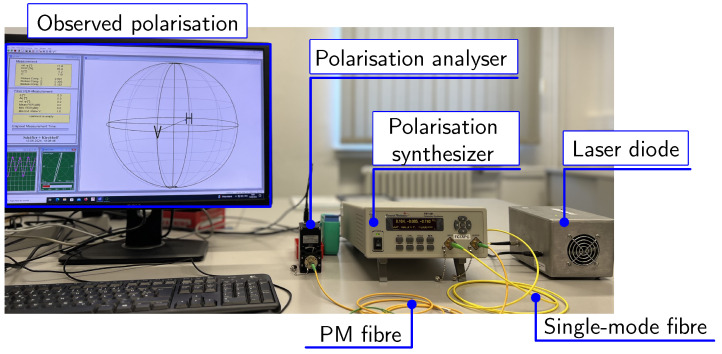
Experimental setup of polarisation-based binary sequence generator with 1m long PMF channel.

In the following step, the output from the combined selection criteria of SEQ_1_ and SEQ_2_ is calculated, which results in the trigger signal. The four-level trigger signal represents four different SOP with their respective amplitudes in accordance with the required threshold levels. This trigger signal is then supplied to the control algorithm of the polarisation synthesizer. The main task of the polarisation synthesizer is to convert a non-polarised light into a desired SOP with the help of a trigger signal. A polarisation-maintaining fibre (PMF) of 1 m is incorporated to propagate the desired SOP. The chosen PMF has an attenuation of 1.0 dB/km. Therefore, the attenuation for a length of a one-meter long PMF will account to $10^{-3}\;\mathrm{dB}/m$. Moreover, the maximum transmit power of the laser diode is −3.68 dBm. Hence, the transmission loss of 1 m long PMF channel is considered to be negligible in the experimental setup. At the end of the transmission channel, a polarisation analyser is incorporated to detect the received SOP. The relative azimuth angle, degree of polarisation (DOP), ellipticity, and Stokes parameters are logged into a text file [39]. Subsequently, an offline signal processing chain is deployed to convert the obtained SOP into the received quantum stream. Finally, a bit stream is calculated from the received quantum stream. A pictorial representation of the experimental setup is shown in Figure 9.

### 3.3. m-Sequences and Gold Sequences

**Figure 10:**
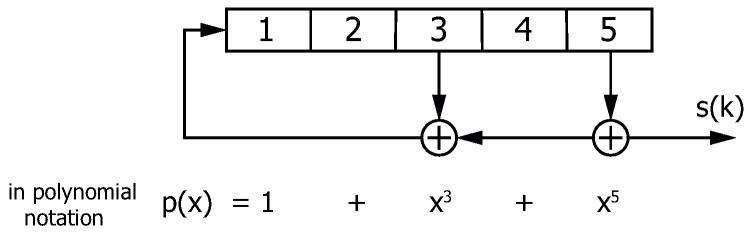
Example for a linear feedback shift register implementation for generating a pseudo-random bit sequence.

A simple class of pseudo-random bit sequences is generated by feedback shift registers as shown in Figure 10.

The output of the linear feedback shift register leads to an *m*-sequence (also known as maximum-length sequence, MLS) if the corresponding generating polynomial p(x) of degree *m* is primitive. The resulting sequence is periodic with the length $L=2^{m}-1$. A further option for generating pseudo-random bit sequences relies on Gold sequences (e.g., [12]). Using two *m*-sequences of the same period *L*, Gold sequences can be obtained when XOR-ing the associated elements of the *m*-sequences.

### 3.4. Cryptographic Primitives

Diffusion and confusion are concepts in cryptography leading to encryption outputs that look more random than regular. A possible candidate is the whole family of Hash functions [7].

With the evolution of the telecommunication industry, the security requirements for exchanging information pose new challenges to cryptography. The main focus is to maintain confidentiality, authentication, and integrity with the help of cryptographic techniques. Cryptographic algorithms are classified into encryption and decryption using symmetric and asymmetric algorithms in which confidentiality is ensured by encrypting the message to be sent by an algorithm with a secret key [40]. Moreover, another category of cryptographic algorithms is also known as cryptographic hash functions in which an arbitrary length of input string is mapped on a fixed length of output string by using mathematical computation [41].

In cryptographic hash functions, an input to the hash function is referred to as a message, and the output of the hash function is defined as a digest. The main property of hash functions is collision-resistant, which implies that the digests from two different messages should not be equal. Additionally, the properties should also include pre-image resistance and second pre-image resistance, which state that it should be computationally impossible to map an input message to a chosen hash value and find a second input with the same hash value, respectively [41]. The merit of using hash functions is that they encrypt the message directly into a digest without the need for a secret key. Table 2 shows the length of output digests for different popular cryptographic hash algorithms.

## 4. Criteria for the Evaluation of Bit Sequences

Randomness in binary sequences has attracted intensive research activities, e.g., [9]. A promising approach for evaluating the properties of binary sequences further was proposed by Goh and Barabasi [42] taking the first and second-order central moments of the gap distribution into account. By taking the mean value $m_{1}$ (average gap length between two neighbouring non-zero elements) as well as the standard deviation σ of the gap lengths, the level of burstiness *B* is obtained as

(9) $$B=\frac{\sigma -m_{1}}{\sigma +m_{1}}\;.$$

When analysing (9), a value B=−1 is obtained for any $m_{1}$ if σ=0 and describes a completely regular (deterministic) process. In this case the density function degenerates to a discrete line at $m_{1}$, and the whole process becomes deterministic. On the other hand, B=0 is considered as a neutral process, as here $\sigma =m_{1}$ holds. Bursty processes appear for 0≤B<1, whereas the parameter B=1 can only be obtained for $m_{1}=0$ for any σ. However, in the case of non-negative random variables (i.e., gaps), the parameter $m_{1}=0$ appears just when all values are equal to zero (i.e., an average gap length of zero). Therefore, B=1 cannot be obtained practically.

Taking the gap density function v(k) (8) into account, the mean value $m_{1}$ and the standard deviation σ can be calculated as follows

(10) $$m_{1}=\sum_{k=0}^{\infty }k\;v\left(k\right)\;\mathrm{and}\;\sigma =\sqrt{\sum_{k=0}^{\infty }k^{2}\;v\left(k\right)-m_{1}^{2}}\;$$

and result in the burstiness level *B* defined in (9).

It is interesting to note that the reciprocal value $1/p_{e}$ of the BOP $p_{e}$ can be calculated from the mean gap distance $m_{1}$ as

(11) $$\frac{1}{p_{e}}=m_{1}+1$$

since the interval $m_{1}+1$ contains a single non-zero bit by definition. With $p_{e}=0.5$, a mean gap distance of $m_{1}=1$ is theoretically expected.

## 5. Results

### 5.1. Introduction

In the following, differences between the various algorithms with regard to the gap density function and the burstiness level *B* will be worked out. The ideal binary sequence defined in (4) can be transferred with the inverse transform method (ITM) [37] in a bit stream and acts as a reference source.

### 5.2. Results from the All-Optical Setup

**Figure 11:**
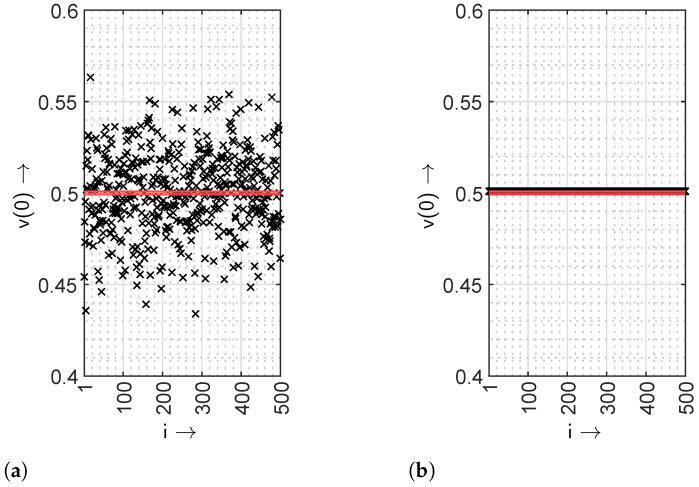
Values v(0) of the output bit stream from the optical measurement setup using different characteristics of SEQ_1_ and SEQ_2_ with *i* instances, where the red line indicates the IID property. (**a**) SEQ_1_ and SEQ_2_ with uniformly distributed pseudo-random binary numbers. (**b**) SEQ_1_ is replaced with an *m*-sequence.

The generated bit sequence from the measurement setup can exhibit various characteristics depending upon the chosen sequences SEQ_1_ and SEQ_2_. For instance, the values of gap density functions for a zero-gap length, i.e., v(0) with two different scenarios are illustrated in Figure 11.

The values v(0) are plotted with 500 instances (realizations) using a sequence length of 1024 for each realization. In Figure 11a, uniformly distributed pseudo-random binary numbers are selected for both of the sequences. It is evident from the results of the first scenario that the IID assumption is obeyed for a few instances only. However, an IID property can be achieved by deploying an *m*-sequence only on SEQ_1_, which is shown in Figure 11b. The overlap of v(0) with the IID characteristic in the red colour for all the instances clearly shows that the IID characteristics of the generated bit sequence can be guaranteed.

**Figure 12:**
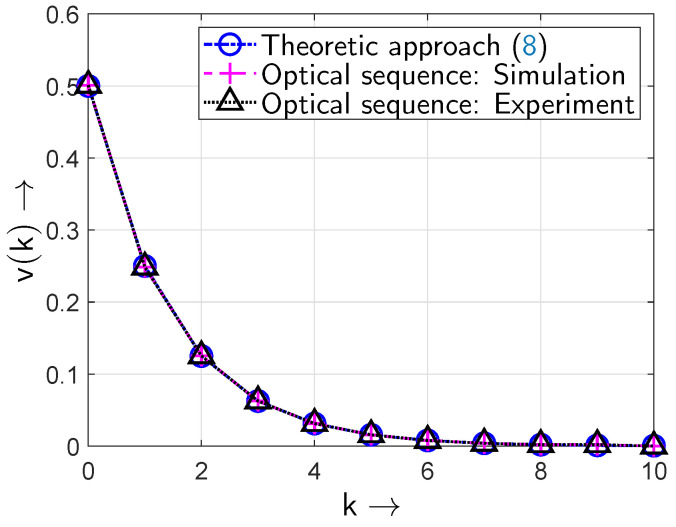
Gap density function v(k) of the optical sequences: simulation and experimental setup (BOP of $p_{e}=0.5$).

According to Figure 12 the results from the experimental setup confirm the proof-of-concept of the all-optical setup and its simulation results and even the theoretically expected values of the gap density function v(k).

### 5.3. Results from Cryptographic Primitives

**Figure 13:**
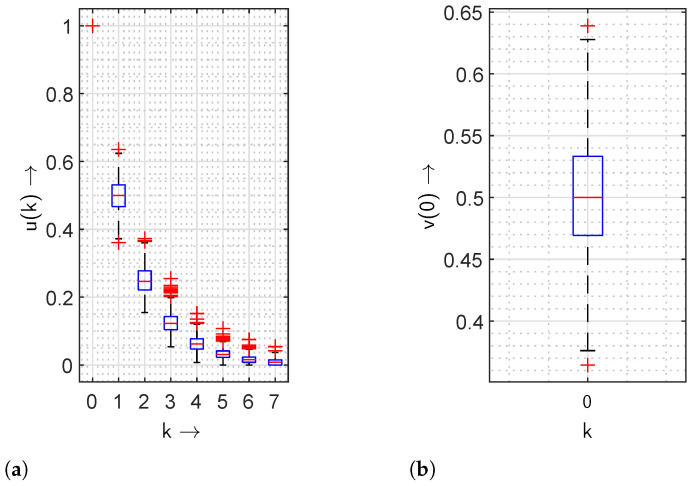
Statistical visualisation of the gap distribution function u(k) and the gap density function v(k) analysing the SHA-256 algorithm using box plots. (**a**) Gap distribution function u(k). (**b**) Gap density function value v(0) of zero gap length.

In Figure 13, values of the gap distribution function and the gap density functions are analysed on the secure hash algorithm, namely SHA-256, with 1000 different hashed values. The gap distribution function and the gap density function of zero gap length are illustrated in Figure 13a and Figure 13b, respectively. The red plus symbols and straight red lines in the box plot represent the outliers and median values, respectively. The 75th percentiles of the u(k) are demonstrated with the help of the area above the median red line. Similarly, the rest of 25th percentiles of the u(k) are shown by the area below. The lines extending from the minimum value to the 25th percentile and from the maximum value to the 75th percentile for all u(k) are the whiskers of each box plot. The gap distribution function is similar to an exponentially decreasing function, which relates to the ideal characteristics of a gap distribution function for independent events or elements, e.g., bits. Moreover, the presence of a cumulative probability less than 0.1% for gaps greater than four corresponds to an improved encryption.

**Figure 14:**
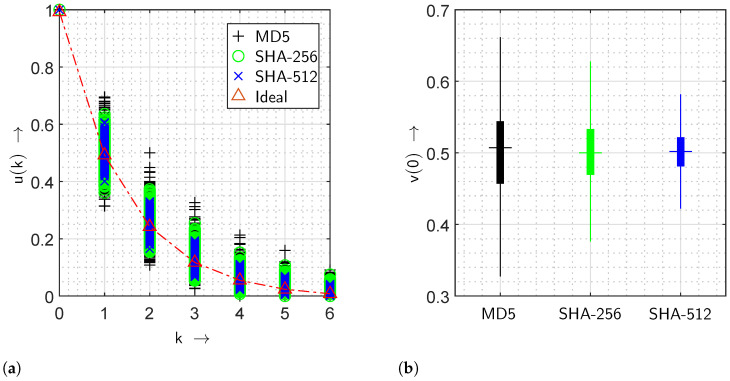
Values of the gap distribution function u(k) and the gap density function v(k) of three different hash algorithms. (**a**) Gap distribution functions u(k) for different algorithms. (**b**) Gap density function value v(0) of zero gap length.

This work is focused on analyzing the gap density function and the gap distribution function of the cryptographic hash functions, which includes currently deployed secure hash algorithms (SHA) such as SHA-256 and SHA-512, and message digest 5 (MD5). From the algorithms mentioned in Table 2, the highest security strength is achieved by SHA-512, followed by SHA-256 and MD5 [43,44]. In Figure 14, gap distribution functions and gap density functions of zero gaps are illustrated with 1000 different digests, which are generated from MD5, SHA-256, and SHA-512 hash algorithms. From Figure 14a, an ideal gap distribution function is calculated using (4), depicted with a red dashed-dotted line. The resulting gap distribution functions of the mentioned hash algorithms converge towards the ideal gap distribution function. The rate of convergence of obtained gap distribution functions corresponds to the security strength for all k>0. It implies that the gap distribution function spread of the MD5 algorithm is the largest due to its lowest security strength, which is overlapped with the gap distribution functions of SHA-256 and on the top of SHA-512. In Figure 14b, the gap density functions of MD5, SHA-256, and SHA-512 algorithms are depicted with k=0 (i.e., zero gap). The whisker length and inter-quartile range of the box-plot approach the IID assumption while utilizing advanced hash algorithms such as SHA-256 and SHA-512 over MD5 algorithms. It also implies that shorter whisker lengths and inter-quartile ranges correspond to a more robust security strength. In conclusion, the gap density function and the gap distribution function are related to the security strength of the cryptographic hash functions, and they provide additional analysis, which can prove beneficial in improving and quantifying the performance of hash algorithms.

### 5.4. Results from Deterministic Algorithms

**Figure 15:**
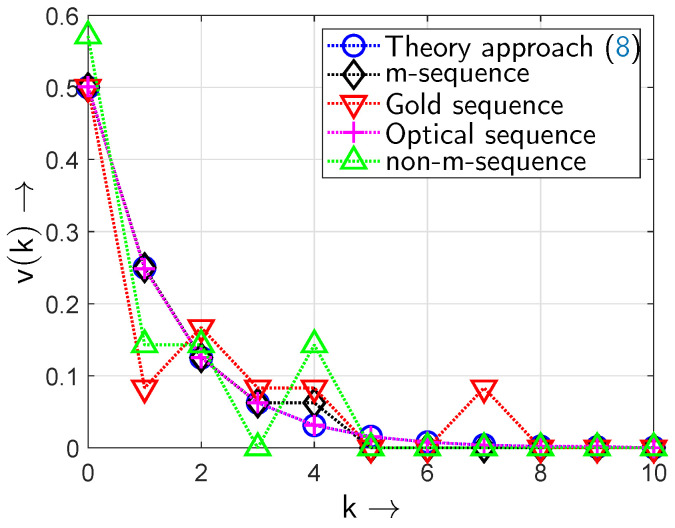
Gap density function v(k) of different sequences (period $L=2^{5}-1=31$; BOP of $p_{e}=0.5$).

Figure 15 shows the gap density functions v(k) for different random sequences. For all sequences a polynomial order of m=5 was chosen resulting in a period of $L=2^{5}-1=31$ for the *m*-sequences. The length of the investigated sequences was set to n=5000 taking a periodisation of the initially calculated *m*-sequence into account. In conjunction with the period *L* this leads to a periodic repetition of the bit patterns in the generated *m*- or Gold sequences.

The *m*-sequence with the generator polynomial $x^{5}+x^{2}+1$ shows gap lengths *k* of 0,1,2,3,4—longer gaps do not occur because of the structure of the sequence. The theoretic formulation of the gap density function in (8) reproduces the behaviour for a gap length of 0…3—but then also accommodates longer gaps (k>4), which are non-existent in the real *m*-sequence. The probabilities for k≥4 in (8) are cumulated in the probability value for k=4 in the measured (simulated) *m*-sequence gap density curve.

The Gold sequence obtained by combining the two *m*-sequences with generator polynomials $x^{5}+x^{2}+1$ and $x^{5}+x^{3}+x^{2}+x+1$, respectively, shows gaps of lengths k=0,1,2,3,4 and k=7. However, gap lengths of k=5,6 occur with zero probability.

The gap density function of the optical sequence shows a characteristic that is approximated very well by the theoretic gap density function (8). Thus, the all-optical setup generates random binary sequences that follow the targeted theoretic gap density curve very well.

For comparison, a sequence with a generator polynomial $x^{5}+x^{4}+x^{3}+1$ has been investigated that is not of maximum length (i.e., it is not an *m*-sequence): here, the gap lengths are also in a range from k=0 to k=4, however, their probabilities differ from those of the *m*-sequence and hence they are not well approximated by (8). In particular, the probability v(0) that a gap of length k=0 occurs is v(0)=0.57. This means that the zero and non-zero elements are not uniformly distributed in such a sequence. Also, for k=3 the probability is v(3)=0, i.e., gaps of length k=3 are not observed.

For k=0 in all cases—except the non-*m*-sequence case—there is the probability of v(0)=0.5 observed: this value describes the probability that a gap of length k=0 occurs; i.e., the probability for a ‘1’ occurring after a ‘1’: this is the BOP $p_{e}$.

**Figure 16:**
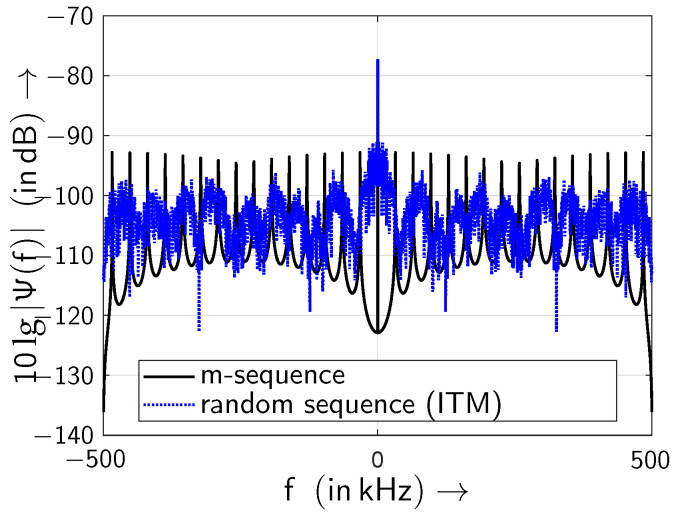
Power spectral densities Ψ(f) of an *m*-sequence and of the sequence generated by using the inverse transform method (sampling frequency $f_{A}=1\;\mathrm{MHz}$).

In order to analyse the periodicity of the investigated sequences, the power density spectrum (PSD) as the Fourier transform of the autocorrelation function is calculated. For comparison, the sampling frequency is set to $f_{A}=1\;\mathrm{MHz}$. In Figure 16 the power spectral density (PSD) Ψ(f) of two investigated sequences are depicted.

First, the *m*-sequence is simulated and the PSD is estimated by autocorrelating the sequence and following Fourier transform: Spectral lines are observed because of the periodicity of the *m*-sequence. Second, a sequence is simulated by generating random numbers representing the gap lengths using the inverse transform method [45] based on the theoretical model (8). In doing so the associated theoretical gap distribution function u(k) according to (4) is inverted for generating the random gap lengths followed by constructing the bit sequence based upon the simulated gap lengths. Using the inverse transform method based on the theoretical gap distribution function u(k), defined in (4), allows for constructing the ideal bit sequence following (4). The gap density function is shown in Figure 15. As this sequence is not periodic, consequently the PSD should change as depicted in Figure 16. In particular, the equidistant spectral lines vanish since the periodicity of the sequence is lost. Hence, the sequence is not a sequence of maximum length and consequently it is not an *m*-sequence.

**Figure 17:**
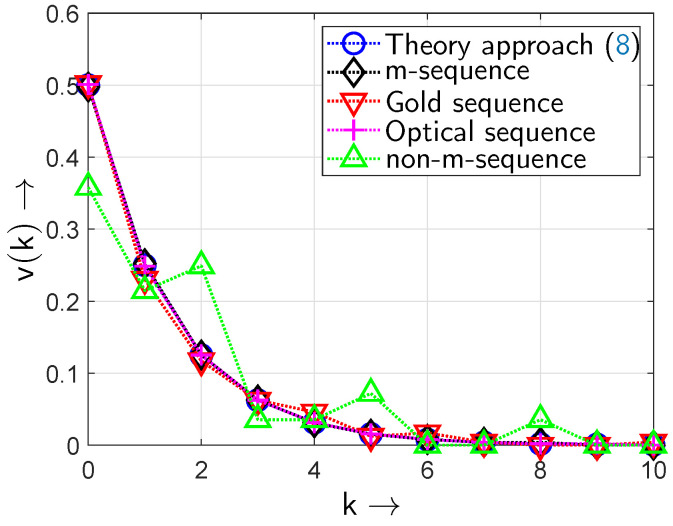
Gap density function v(k) of different sequences (period $L=2^{9}-1=511$; BOP of $p_{e}=0.5$).

Figure 17 shows the gap density functions v(k) for longer sequences: now, polynomials of degree m=9 are considered to lead to sequences of length $L=2^{9}-1=511$. The *m*-sequence has been generated by using the generator polynomial $x^{9}+x^{4}+1$. The Gold sequence is obtained by combining the two *m*-sequences with generator polynomials $x^{9}+x^{4}+1$ and $x^{9}+x^{6}+x^{4}+x^{3}+1$, respectively. For comparison, a sequence with a generator polynomial $x^{9}+x^{6}+x^{3}+x+1$ has been investigated that is not of maximum length (i.e., it is not an *m*-sequence): their probabilities are, again, not well approximated by (8). In particular, the probability v(0) that a gap of length k=0 occurs is v(0)=0.36; again, the zero and non-zero elements are not uniformly distributed in such a sequence.

When comparing the results of Figure 15 and Figure 17 it becomes obvious that greater sequence lengths are preferable when targeting the behaviour of the theoretic curve (8): outliers are minimized and the generated sequences resemble the theoretic curve (8) better in terms of their gap density functions when generator polynomials of higher degrees are used.

### 5.5. Burstiness Level

Finally, the distribution of the zeros and ones in the generated sequences is studied by calculating the corresponding levels of burstiness. In Table 3 the burstiness levels *B* are put together for the different investigated sequences.

All values for *B* are between 0 and 1—as expected and explained in Section 4. In particular, all levels of burstiness are between 0.1 and 0.2—this means that all investigated algorithms usually produce rather non-bursty sequences—as their *B* is closer to 0 (neutral) than to 1 (bursty). When taking a closer look, it is recognizable that the *m*-sequence shows the smallest *B* among the considered sequences and hence shows the most non-bursty result. The other sequences show slightly higher levels of burstiness meaning that their bit sequences are slightly more bursty and the ‘1’ elements occur more in groups (bursts).

In addition, two *m*-sequences with the higher-order m=9 and the associated period $M=2^{9}-1=511$ were investigated in terms of their burstiness. In Table 4 the associated results are compiled.

The results show that the sequence where the non-zero coefficients in the primitive polynomial p(x) are usually rather uniformly distributed shows a lower level of burstiness than in the case where the non-zero coefficients of the primitive polynomial’s elements are more concentrated. Thus, primitive polynomials with more concentrated non-zero coefficients in the primitive polynomial lead to more bursty bit sequences. This is a result that has already been confirmed by the analysis of undetected error structures in coded transmission systems [46].

## 6. Discussion

Many applications require random sequences which fulfil the IID property, e.g., that they are independent and show an identical probability distribution. Under these boundary conditions, the optimal gap distribution function was derived in (4). The IID characteristic implies further that the gap lengths within a pseudo-random sequence are independent from each other. Under these circumstances the gap distribution function is solely described by the BOP.

The condition $v\left(0\right)=p_{e}$ can be guaranteed for an independent distribution of the “1” elements within the given binary sequence. Under bursty conditions, i.e., the “1” elements in the sequence occur more in groups (see Figure 3 and Figure 6), the value v(0)>0.5 is observed at the cost that the probability of longer gaps decreases. In this situation, (4) is no longer suited to describe the distribution of the gaps and gap distribution functions with two or more parameters are suited such as the Weibull distribution [37] or distributions introduced by Wilhelm [33].

As the investigated gap approaches are based on the independency of the gap lengths, the distribution of the zeros and ones within the random sequence gains additional interest. That is why the burstiness levels were calculated in this work based on Goh and Barabasies’ approach. The obtained levels confirm a rather non-bursty distribution of the one elements within a given sequence. In this respect the use of (4) can be justified.

The simulation of sequences generated by feedback shift registers, e.g., to generate *m*-sequences, have shown that they can only get close to the ideal distribution for large periods *L*. The all-optical setup represents an alternative approach for generating pseudo-random sequences, which can adapt to the properties of an input sequence. For instance, an IID sequence is achieved by the all-optical setup by using an *m*-sequence as SEQ in the all-optical setup. Therefore, the desired characteristics of the output binary sequences can be tailored with the help of the first sequence.

In this work, the security strength of cryptographic primitives is also analysed using the gap-based approach and gap distribution function. From the results, it is evident that the gap distribution function of the output digest of the cryptographic algorithm is converging towards an ideal gap distribution function with the increase in the security strength of the advanced cryptographic algorithms. Moreover, the zero-gap gap density function of the higher security strength algorithm is observed to approach an IID characteristic. The values of the output digest of SHA-512 are situated closer to the IID point in comparison to the MD5 algorithm. Therefore, gap density function and gap distribution function provide an alternative to access the security strength of the cryptographic primitives.

## 7. Conclusions

In this article, different methods for generating random binary sequences are investigated and compared. Such sequences have important applications in communications engineering, e.g., for creating random bit sources in a simulation context, or in the field of cryptography, e.g., for establishing the basis of cryptographic keys. The relation between the security strength of a cryptographic hash function and the proposed gap distribution function is established, which provides an objective understanding about the robustness of hash functions. Classic approaches like *m*-sequences and Gold sequences are simulated together with random binary sequences without their special characteristics (non-*m*-sequences). Furthermore, in particular the more recent advances of the all-optical generation of random binary sequences have been taken into account as well and they are also simulated, practically evaluated, and compared with the other approaches. The results show very good agreement between the all-optically generated sequences and the theoretical approach in terms of their probability density functions. Thus, they are very good candidates when it comes to random sequences with desirable properties. A criterion *burstiness level* is used to compare the different sequences in terms of their bursty or non-bursty behaviour. Here, it turned out that in particular the *m*-sequences ensure a non-bursty characteristic among the considered sequences. Also, the more the non-zero elements of the generator polynomial are uniformly distributed, the more the generated random sequences are non-bursty, i.e., their elements are more independently distributed. The proposed all-optical setup is an alternative method to generate the binary sequences with desired characteristics. These sequences resemble the properties given by the theoretical gap distribution function (4) very well. Thereby, the proposed experimental optical setup confirms the all-optical simulation results.

## Figures and Tables

**Table 1:** Generation of trigger signal according to the SEQ_1_ and SEQ_2_.

Types of Polarisation	SEQ_1_ Select	SEQ_2_ Select
Horizontal	0	0
Vertical	1	0
Right circular	1	1
Left circular	0	1

**Table 2:** Length of output digests for different cryptographic hash algorithms.

Cryptographic Hash Algorithms	Length of Digest (in Bits)
MD5	128
SHA-256	256
SHA-512	512

**Table 3:** Burstiness levels *B* of different sequences.

*m*-Sequence	Sequence (ITM)	Gold Sequence	Optical Sequence	Non-*m*-Sequence
0.1218	0.1846	0.1408	0.1689	0.1717

**Table 4:** Burstiness levels *B* of two *m*-sequences (*m* = 9).

Primitive Polynomial *p*(*x*)	Burstiness Level *B*
$x^{9}+x^{4}+1$	0.1641
$x^{9}+x^{8}+1$	0.1777

## Data Availability

Data are contained within the article.

## Abbreviations

The following abbreviations are used in this manuscript:

BOP	Bit occurrence probability
DOP	Degree of polarisation
IID	Independently and identically distributed
ITM	Inverse transform method
LD	Laser diode
MLS	Maximum length sequence
MMF	Multi-mode fibre
NIST	National Institute of Standards and Technology
PMF	Polarisation-maintaining fibre
PSD	Power spectral density
QKD	Quantum key distribution
SEQ	Sequence
SMF	Single-mode fibre
SOP	State of polarisation
VOA	Variable optical attenuator
